# Tolerance to high-intensity intermittent running exercise: do oxygen uptake kinetics really matter?

**DOI:** 10.3389/fphys.2012.00406

**Published:** 2012-10-22

**Authors:** Martin Buchheit, Karim Hader, Alberto Mendez-Villanueva

**Affiliations:** Physiology Unit, Football Performance and Science Department, ASPIRE Academy for Sports ExcellenceDoha, Qatar

**Keywords:** metabolic recovery, time constant, electromyography, near-infrared spectroscopy, team sports

## Abstract

We examined the respective associations between aerobic fitness ($\dot{V}{\text{O}}_{\text{2}}$max), metabolic control ($\dot{V}{\text{O}}_{\text{2}}$ kinetics) and locomotor function, and various physiological responses to high-intensity intermittent (HIT) running exercise in team sport players. Eleven players (30.5 ± 3.6 year) performed a series of tests to determine their $\dot{V}{\text{O}}_{\text{2}}$max and the associated velocity (v$\dot{V}{\text{O}}_{\text{2}}$max), maximal sprinting speed (MSS) and $\dot{V}{\text{O}}_{\text{2}}$ kinetics at exercise onset in the moderate and severe intensity domains, and during recovery ($\dot{V}O_{2}\tau_{\text{off}}$ SEV). Cardiorespiratory variables, oxygenation and electromyography of lower limbs muscles and blood lactate ([La]) concentration were collected during a standardized HIT protocol consisting in 8 sets of 10, 4-s runs. During HIT, four players could not complete more than two sets; the others finished at least five sets. Metabolic responses to the two first sets of HIT were negatively correlated with $\dot{V}{\text{O}}_{\text{2}}$max, v$\dot{V}{\text{O}}_{\text{2}}$max, and $\dot{V}O_{2}\tau_{\text{off}}$ SEV (*r* = −0.6 to −0.8), while there was no clear relationship with the other variables. $\dot{V}{\text{O}}_{\text{2}}$, oxygenation and [La] responses to the first two sets of HIT were the only variables that differed between the players which could complete at least five sets or those who could not complete more than two sets. Players that managed to run at least five sets presented, in comparison with the others, greater v$\dot{V}{\text{O}}_{\text{2}}$max [ES = +1.5(0.4; 2.7), MSS(ES = +1.0(0.1; 1.9)] and training load [ES = +3.8 (2.8; 4.9)]. There was no clear between-group difference in any of the $\dot{V}{\text{O}}_{\text{2}}$ kinetics measures [e.g., ES = −0.1(−1.4; 1.2) for $\dot{V}O_{2}\tau_{\text{on}}$ SEV]. While $\dot{V}{\text{O}}_{\text{2}}$max and v$\dot{V}{\text{O}}_{\text{2}}$max are likely determinant for HIT tolerance, the importance of $\dot{V}{\text{O}}_{\text{2}}$ kinetics as assessed in this study appears limited in the present population. Knowing the main factors influencing tolerance to HIT running exercise may assist practitioners in personalizing training interventions.

## Introduction

Time motion match analyses have provided important information on the physical demands of team sports (Ben Abdelkrim et al., 2007; Di Salvo et al., 2007; Buchheit et al., 2010b; Povoas et al., 2012), with the most determinant actions generally occurring following (repeated) high-intensity actions (Faude et al., 2012). While this might be position-, team formation- and individual playing style-dependent (Buchheit et al., 2010b; Mendez-Villanueva et al., 2011b), the ability to perform and tolerate HIT efforts is therefore believed to be an important fitness prerequisite at the elite level.

Tolerance to HIT running exercise is believed to be related to several physiological and locomotor attributes such as cardiorespiratory fitness (maximal oxygen uptake, $\dot{V}{\text{O}}_{\text{2}}$max), the speed associated with maximal oxygen uptake (v$\dot{V}{\text{O}}_{\text{2}}$max) (Dupont et al., 2010a), the ability to quickly activate the aerobic system [as measured by the time constant (τ) of $\dot{V}{\text{O}}_{\text{2}}$ kinetics] (Dupont et al., 2005; Rampinini et al., 2009; Dupont et al., 2010b), and acceleration capacity and explosive strength of the lower limbs (Buchheit, 2008). For a given absolute running speed, a high v$\dot{V}{\text{O}}_{\text{2}}$max is responsible for reduced relative exercise intensity (Mendez-Villanueva et al., 2012), thereby delaying fatigue and improving exercise tolerance. Fast $\dot{V}{\text{O}}_{\text{2}}$ kinetics at exercise onset may also prevent excessive local peripheral physiological disturbance, thereby sparing anaerobic capacity, in turn allowing for the maintenance of high-intensity running capacity during subsequent interval bouts (Jones and Burnley, 2009). Finally, rapid $\dot{V}{\text{O}}_{\text{2}}$ off-kinetics appear related to faster post-efforts replenishment of muscle O_2_ and phosphocreatine (PCr) stores (Borsheim and Bahr, 2003), which likely improves successive high-intensity exercise tolerance (Girard et al., 2011).

While the physiological rationale for the expected relationships between on- and off-$\dot{V}{\text{O}}_{\text{2}}$ kinetics and HIT running tolerance makes intuitive sense [i.e., the lower the O_2_ deficit and the faster the metabolic recovery, the better the high-intensity running capacity (Girard et al., 2011)], research findings have been inconclusive (Dupont et al., 2005; Rampinini et al., 2009; Dupont et al., 2010b; Buchheit, 2012a,b; Buchheit et al., 2012a). While in soccer players, large to very-large correlations were reported between repeated-sprint ability and on- (Dupont et al., 2005; Rampinini et al., 2009) and off- (Dupont et al., 2010b) $\dot{V}{\text{O}}_{\text{2}}$ kinetics, Buchheit et al. could not find any relationship between repeated-sprint ability and $\dot{V}{\text{O}}_{\text{2}}$ kinetics in moderately-trained cyclists (Buchheit et al., 2012a). This lack of association was confirmed in a subsequent study involving 61 team sport players (Buchheit, 2012b). In this latter study, the locomotor profile of the players (i.e., maximal aerobic and sprinting speeds) was the exclusive predictor of repeated-sprint performance (stepwise regression analysis). “Metabolic” variables such as $\dot{V}{\text{O}}_{\text{2}}$max and on- and off-$\dot{V}{\text{O}}_{\text{2}}$ kinetics were excluded from the statistical model (Buchheit, 2012b).

The lack of agreement on the relative importance of $\dot{V}{\text{O}}_{\text{2}}$ kinetics for high-intensity running performance may be related to differences in study populations and methodological considerations. It was first suggested that the previously reported associations between repeated-sprint ability and $\dot{V}{\text{O}}_{\text{2}}$ kinetics (Dupont et al., 2005; Rampinini et al., 2009; Dupont et al., 2010b) may not reflect a cause-and-effect mechanism, but instead could be related to the particular fitness profile of the soccer players examined in these studies (Buchheit, 2012b). For instance, in this specific (young) population, a large correlation exists between maximal sprinting speed (MSS) and maximal aerobic function (Mendez-Villanueva et al., 2010) [to which $\dot{V}{\text{O}}_{\text{2}}$ kinetics are generally related (mediated by players' training status) (Kilding et al., 2006)]. Additionally, since in team sport players, performance during repeated-sprint sequences (i.e., all-out maximal efforts) is almost exclusively determined by neuromuscular factors [i.e., springing speed (Mendez-Villanueva et al., 2011a; Buchheit, 2012b)], the metabolic component of repeated-sprint ability that could be affected by $\dot{V}{\text{O}}_{\text{2}}$ kinetics may not be important enough during some sequences to substantially impact repeated-sprint performance. In these lines, $\dot{V}{\text{O}}_{\text{2}}$ kinetics could be more important for high-intensity (but not obligatory maximal) intermittent running tolerance/performance, which is likely more related to metabolic (Rampinini et al., 2008; Dupont et al., 2010a) than neuromuscular factors. Finally, in the two studies by Buchheit et al. (2012a) and Buchheit (2012b), $\dot{V}{\text{O}}_{\text{2}}$ kinetics were assessed during transitions from rest to moderate-intensity exercise, which differed from that reached during the repeated-sprint exercises examined (i.e., supramaximal intensity).

The impact that on- and off-$\dot{V}{\text{O}}_{\text{2}}$ kinetics, measured in the severe intensity domain, have on HIT running tolerance is therefore still unknown. This has also probably greater practical implications than the previously examined relationships with repeated-sprint performance (Dupont et al., 2005; Rampinini et al., 2009; Dupont et al., 2010b; Buchheit, 2012b; Buchheit et al., 2012a), since in contrast to the important high-intensity running demands during games (Ben Abdelkrim et al., 2007; Di Salvo et al., 2007; Buchheit et al., 2010b; Povoas et al., 2012), the actual occurrence of repeated-sprint sequences is quite low [at least in elite adult and highly-trained young soccer payers (Buchheit et al., 2010c; Carling et al., 2012)]. Therefore, to improve our understanding of the physiological and locomotor factors affecting tolerance to HIT running exercise, we compared the respective impact of selected measures of anaerobic and aerobic fitness, metabolic control and locomotor function on players' tolerance to a standardized HIT running protocol. We expected to observe negative correlations between the aforementioned individual capacities and both metabolic responses and neuromuscular impairments following the HIT running protocol. However, due to the contradictory findings in the literature and the lack of studies on this particular exercise modality, the magnitude of these correlations was difficult to predict. In practice, a better understanding of the factor affecting tolerance to HIT efforts is key to structure and individualize training programs in team sport players.

## Methods

### Participants

Eleven recreational team sport players (30.5 ± 3.6 year, 81 ± 6 kg, 180 ± 6 cm) volunteered for this study. They were all involved (4.9 ± 2.7 h·week-1) in soccer, handball or Australian Rules Football and had no history or clinical signs of cardiovascular or pulmonary diseases. Participants were not currently taking prescribed medications and presented with normal blood pressure levels and electrocardiographic patterns. All players gave voluntary written consent to participate in the experiment. The study conformed to the recommendations of the Declaration of Helsinki and was approved by the local Review Board for use of Human Subjects.

### Experimental overview

**Figure 1:**
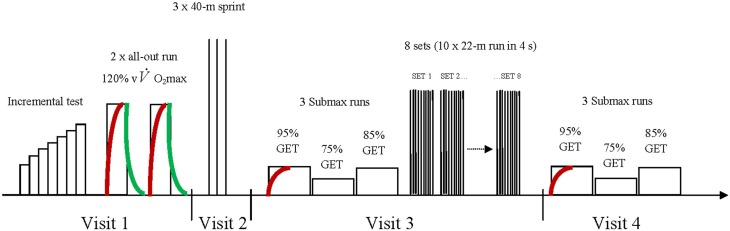
**Schematic representation of the protocol timing during the four visits.** v$\dot{V}{\text{O}}_{\text{2}}$max, running speed associated with maximal oxygen uptake; GET, gas exchange threshold. The red and green curves represent $\dot{V}{\text{O}}_{\text{2}}$max kinetics assessments at exercise onset and cessation, respectively. See “Methods” for more details on within-day timings.

All players were familiarized with all testing procedures before the start of the experimentations. Each player was tested on four occasions, separated by at least 48 h (Figure 1). The first session consisted of two different series of tests. Players first performed an incremental running test to determine maximal oxygen uptake ($\dot{V}{\text{O}}_{\text{2}}$max) and the associated velocity (v$\dot{V}{\text{O}}_{\text{2}}$max). Then, 10 min following the incremental test, they performed two supramaximal runs until exhaustion at 120% of v$\dot{V}{\text{O}}_{\text{2}}$max (interspersed by 10 min of passive recovery) to determine the kinetics of $\dot{V}{\text{O}}_{\text{2}}$ in the severe intensity domain both at exercise onset and cessation. During the second session, after a standardized warm-up, players performed three 40-m sprints to determine their MSS. During the third session, players performed three submaximal 5-min run (with the first used to determine the kinetics of $\dot{V}{\text{O}}_{\text{2}}$ at exercise onset in the moderate-intensity domain), and then, a standardized HIT running exercise. Finally, players performed three additional submaximal runs during a fourth visit (Figure 1). Cardiorespiratory variables, oxygenation (Near-infrared spectroscopy measurements, NIRS) and electromyography (EMG) of lower limbs muscles, blood lactate ([La]) and rating of perceived exertion (RPE) (0–10 on Borg's scale) were collected for all tests. Players also performed two counter movement jumps (CMJ) and two drop jumps (DJ) before and after the two first running sets of the HIT exercise. All tests were performed on an indoor synthetic track where ambient temperature ranged from 18°C to 22°C. Subjects were told not to perform exercise on the day prior to a test, and to consume their last (caffeine free) meal at least 3 h before the scheduled test time.

## Exercise protocols

### Incremental field running test

A modified version of the University of Montreal Track Test [UM-TT, (Leger and Boucher, 1980)] (i.e., Vam-Eval) was used to determine maximal oxygen uptake ($\dot{V}{\text{O}}_{\text{2}}$max) and the associated running velocity (v$\dot{V}{\text{O}}_{\text{2}}$max, see “Data Collection and Analyses” paragraph). The Vam-Eval is very similar to the UM-TT, i.e., same speed increments. The only difference between the two tests is the distance between the cones placed along the athletic track [i.e., 20 (Vam-Eval) vs. 50 (Um-TT) m], which renders the Vam-Eval easier to administer. The test began with an initial running speed of 8.5 km.h^−1^ with consecutive speed increases of 0.5 km.h^−1^ each minute until exhaustion. The players adjusted their running speed according to auditory signals timed to match 20-m intervals delineated by marker cones around a 200-m long indoor athletics track. Throughout the test, players were given verbal encouragement by the testers and coaches. The test ended when the players failed on two consecutive occasions to reach the next cone in the required time.

### Maximal sprinting speed

All players performed three maximal 40-m sprints during which 10-m split times were recorded using dual-beam electronic timing gates (Swift Performance Equipment, Lismore, Australia) (Figure 1). MSS was defined as the fastest 10-m split time measured during a maximal 40-m sprint (Buchheit et al., 2012b). Split times were measured to the nearest 0.01 s. Players commenced each sprint from a standing start with their front foot 0.5 m behind the first timing gate and were instructed to sprint as fast as possible over the full 40 m. The players started when ready, thus eliminating reaction time. Each trial was separated by at least 60 s of recovery with the best performances used as the final result. The reliability of MSS was assessed prior to the present study in a group of 65 young soccer players: the typical error, expressed as a coefficient of variation (CV), was 1.4% (1.2; 1.6).

### Anaerobic speed reserve

The anaerobic speed reserve (ASR) was calculated as follow (Mendez-Villanueva et al., 2012):

$$\text{ASR}\left(\text{km}.{\text{h}}^{-\text{1}}\right)=\text{MSS}-\text{v}\dot{V}{\text{O}}_{\text{2}}\text{max}$$

### Moderate-intensity runs

During both the pre-HIT session warm-up and the fourth testing session, players performed three consecutive submaximal runs (Figure 1). Before the first run, player rested (standing) passively for 2 min, and then ran at an intensity corresponding to 95% of their gas exchange threshold (GET) as measured during the incremental test (see “Data Collection and Analyses” paragraph) (Figure 1), which is well suited to determine the $\dot{V}{\text{O}}_{\text{2}}$ kinetics in the moderate-intensity domain (Whipp et al., 2005) and allowed for comparisons with previous studies examining the relationship between repeated-sprint performance and $\dot{V}{\text{O}}_{\text{2}}$ on-kinetics (Dupont et al., 2005; Rampinini et al., 2009; Buchheit, 2012b; Buchheit et al., 2012a). The two following runs were performed consecutively at 75% and 85% of GET, respectively. Taken together, these three submaximal runs were used to derive the individual $\dot{V}{\text{O}}_{\text{2}}$/speed relationship for each player [see paragraph on “Maximal Accumulated O_2_ Deficit (MAOD)”]. In the field, an audio time countdown was given to the subjects 3 s before the commencement of the test. Running pace was governed by a prerecorded beep that sounded at appropriate intervals to allow participants to adjust their running speed as they passed through specific zones of the track (i.e., a cone placed every 20 m). Particular attention was focused on ensuring that the player reached the required running speed within at least 5 s [participants had to be near an additional cone, placed 10–12 m (depending on their running speed) from the starting line; i.e., within 5 ± 1 s]. If adjustment to the required running speed was not satisfactory (i.e., subjects passed a cone outside of a 1-s difference compared with expected time), the test was stopped, and the subject was asked to recommence the test after a 5-min period of passive recovery.

### Supramaximal runs

Ten min after the incremental test, participants ran twice to the point of volitional exhaustion at a speed corresponding to 120% of their v$\dot{V}{\text{O}}_{\text{2}}$max (Dupont et al., 2010b). There was a 10-min period of passive recovery between the two runs. Running pace was governed by a pre-recorded beep that sounded at appropriate intervals to adjust running speed as they passed close to visual marks, every 20 m, along the track. Time was measured to the nearest second from the moment the participants gave up or were unable to reach the marks on time on two consecutive occasions. Strong verbal encouragements were given throughout each run.

### Lower limb explosive strength tests

Lower limb explosive strength was assessed using vertical CMJ and DJ (cm) with jump height measured by a force plate (Kistler Instruments, Amherst, Massachusetts). Each type of jump was repeated twice before and after the two first running sets of the HIT exercise (see below). The participants were instructed to keep their hands on their hips during both CMJ and DJ. For CMJ, the subjects were instructed to dip to their optimal depth from a standing position and immediately jump for maximum height. CMJ were performed in a continuous movement with no pause between downward and upward phases. DJ were executed from a 36-cm box without any leg flexion. Players were requested to minimize ground contact time and to jump as high as possible. Each trial was validated by visual inspection. For both CMJ and DJ, the average jumping height over the two trials at each time point was retained for analysis. Additionally, to account for possible between-player differences in body mass, power output (W) during each jump was also estimated (Sayers et al., 1999).

### High-intensity intermittent running exercise

Upon completion of the three submaximal runs (Figure 1), players performed 3 min of athletic drills (e. g., skipping, high knee runs), five short bursts of progressive accelerations on the track and two 22-m sprints. Following pre-HIT jumps, players completed the HIT protocol consisting of repeating 8 sets of 10, 4-s straight-line runs, departing every 16 s. Sets were interspersed with 2 min 20 s of recovery. The effort sequence during the sets was chosen based on the average HIT effort profile of a soccer game [i.e., 2.2 s/18 s (Vigne et al., 2010) and the work/recovery ratio of the most intense 5-min period 2.2s/13s (Mohr et al., 2003)]. The average speed during each run was 19.8 km.h^−1^, which is generally considered as high-intensity running effort (Mohr et al., 2003; Di Salvo et al., 2007; Buchheit et al., 2010b). Between each run, subjects performed an active running recovery at 6 km.h^−1^. An audio feedback (i.e., time countdown) was given to the subjects so that they maintained the required running speed. Three seconds prior to the commencement of each run, subjects were asked to assume the ready position and await the start signal. They were instructed to complete all runs within the allowed time (i.e., 4 s), and strong verbal encouragement was provided during all runs. While all players were expected to finish the eight sets of the present HIT protocols, the test was terminated earlier if the players failed on two consecutive occasions to complete the 22-m run in the required time.

## Data collection and analyses

### Cardiorespiratory measures and v$\dot{V}{\text{O}}_{\text{2}}$max determination

Respiratory gas exchange and heart rate (HR) were measured using an automated, portable, breath-by-breath system (Oxycon Pro, Carefusion GmbH, Hoechberg, Germany) during all tests. Before each test, the O_2_ and CO_2_ analysis systems were calibrated as recommended by the manufacturer. Cardiorespiratory values were averaged over 5-s periods for all tests. Since the validity of the usual criteria for establishing $\dot{V}{\text{O}}_{\text{2}}$max during ramp exercise tests has been questioned (Poole et al., 2008), $\dot{V}{\text{O}}_{\text{2}}$max was defined as the highest $\dot{V}{\text{O}}_{\text{2}}$ values attained in a 30-s epoch during the incremental test. An inability to maintain the required running speed, high values for blood [La] (>8 mmol.l^−1^) and rate of perceived exertion (>16 on the 6–20 Borg scale) were also required to confirm the maximal nature of the test. Additionally, we were confident in the maximal nature of the tests, since all players were highly motivated and performed until voluntary exhaustion. GET was defined as the speed at which there was a non-linear increase in VE/VO_2_ without a concomitant non-linear increase in VE/VCO_2_. The peak HR (5-s average) reached during the incremental test was retained as maximal HR (HR_max_). The lowest running speed eliciting $\dot{V}{\text{O}}_{\text{2}}$max for at least 30 s was retained as v$\dot{V}{\text{O}}_{\text{2}}$max. The reliability of v$\dot{V}{\text{O}}_{\text{2}}$max is good in moderately-trained middle and long distance runners, with a typical error, expressed as a CV of 3% (Midgley et al., 2007b). Finally, $\dot{V}{\text{O}}_{\text{2}}$ values during HIT were expressed as a percentage of each player's $\dot{V}{\text{O}}_{\text{2}}$max (%$\dot{V}{\text{O}}_{\text{2}}$max) and were averaged during each set to provide a single $\dot{V}{\text{O}}_{\text{2}}$ value per set.

### Assessment of oxygen uptake kinetics

Because of the high variability between breaths, increasing the number of exercise transitions in the same exercise protocol is a common practice used to increase the signal-to-noise ratio (Whipp et al., 2005) and provide the highest possible confidence in the data subsequently modeled. In the present study, players performed two transitions, which is similar to previous studies investigating the relationship between $\dot{V}{\text{O}}_{\text{2}}$ kinetics and repeated-sprint performance (Dupont et al., 2005; Rampinini et al., 2009; Dupont et al., 2010b; Buchheit, 2012b; Buchheit et al., 2012a). The $\dot{V}{\text{O}}_{\text{2}}$ data sets of the two moderate-intensity runs performed at 95% of GET, as well as these from the two supramaximal runs, were then averaged together to produce two unique responses for each subject (one for moderate-intensity runs and one for the supramaximal runs). When the total running time differed between the two consecutive supramaximal runs, exercise data was split and averaged to allow the synchronization of the on- and off- transient kinetics. For example, for $\dot{V}{\text{O}}_{\text{2}}$ on-transient kinetics, $\dot{V}{\text{O}}_{\text{2}}$ data from both exercises were averaged from 2 min prior exercise onset to the end of the shorter exercise (approx. 90–100 s, see “Results”). For $\dot{V}{\text{O}}_{\text{2}}$ off-transient kinetics, $\dot{V}{\text{O}}_{\text{2}}$ data from both exercises were first synchronized at exercise cessation. $\dot{V}{\text{O}}_{\text{2}}$ data were then averaged from 60 s pre exercise cessation to 10 min post; this segment was then retained for analysis.

**Figure 2:**
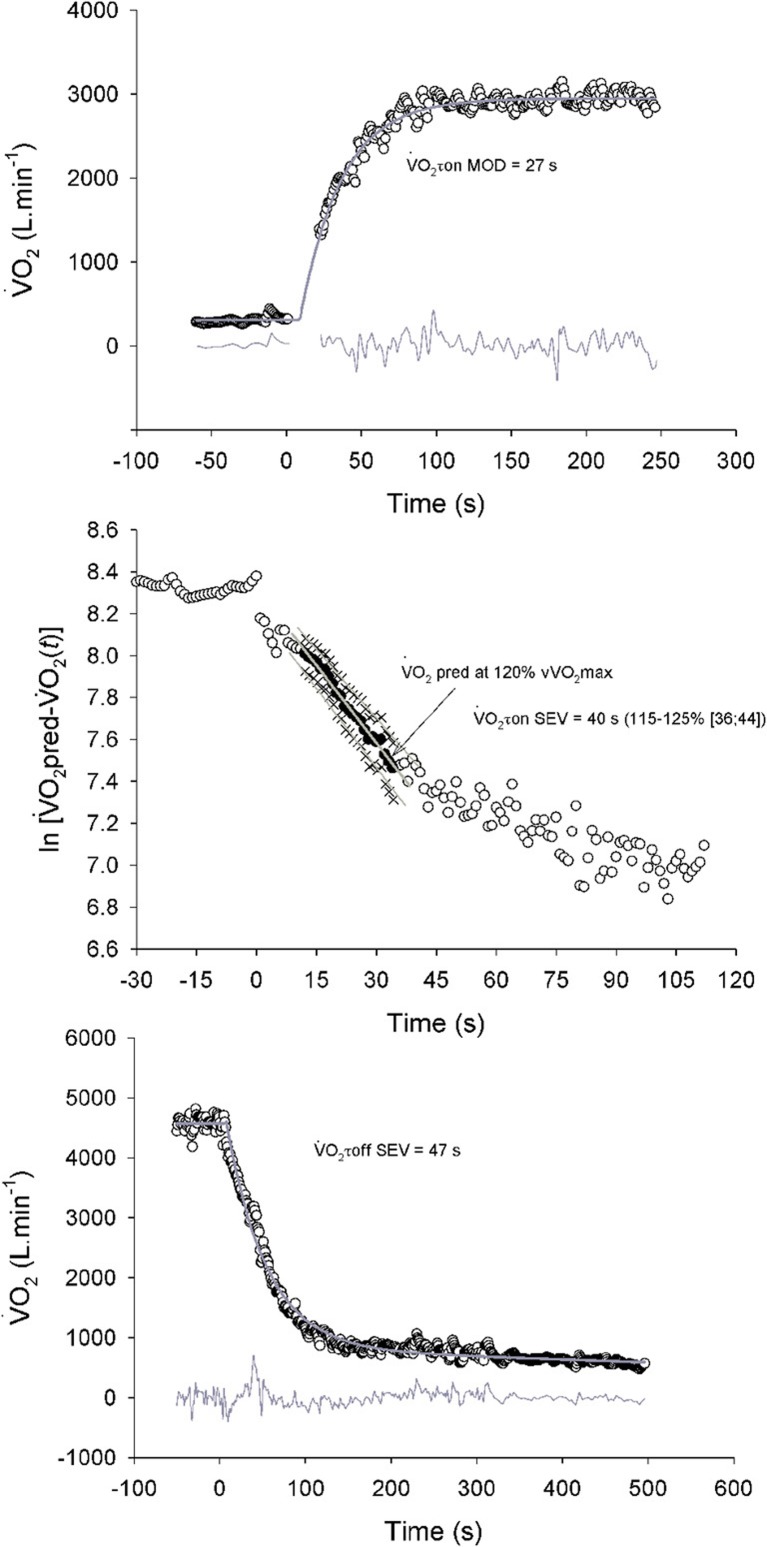
**Example of oxygen uptake ($\dot{V}{\text{O}}_{\text{2}}$) kinetics determination in a representative subject at exercise onset (upper and mid panel) and during exercise recovery (lower panel).** $\dot{V}O_{2}\tau_{\text{on}}$ MOD and $\dot{V}O_{2}\tau_{\text{on}}$ SEV are the time constants of the primary component of the pulmonary $\dot{V}{\text{O}}_{\text{2}}$ kinetics at exercise onset in the moderate and severe exercise intensity domain, respectively. $\dot{V}O_{2}\tau_{\text{off}}$ SEV is the time constants of the primary component of the pulmonary $\dot{V}{\text{O}}_{\text{2}}$ kinetics after exercise performed in the severe intensity domain. The bottom gray line in the upper and lower panels represents the residuals.

For the moderate-intensity exercise, $\dot{V}{\text{O}}_{\text{2}}$ on-transient kinetics were modeled using an iterative technique (Sigmaplot 10, SPSS Science; Chicago, IL, USA) using either a mono- (Equation 1) or a bi-exponential function (Equation 2) (Figure 2, upper panel):

(1) $$\dot{V}{\text{O}}_{\text{2}}\left(t\right)=\dot{V}{\text{O}}_{\text{2}}+\text{Ampl}\times \left[\text{1}-{\text{e}}^{-\left(\text{t}-\text{TD}/\dot{V}\text{O2}\tau \text{on}\right)}\right]\times U_{\text{1}}$$

(2) $$\dot{V}{\text{O}}_{\text{2}}\left(t\right)=\dot{V}{\text{O}}_{\text{2}}+\text{Ampl}\times \left[\text{1}-{\text{e}}^{-\left(\text{t}-\text{TD}/\dot{V}\text{O2}\tau \text{on}\right)}\right]\times U_{\text{1}}+{\text{Ampl}}_{\text{2}}\times \left[\text{1}-{\text{e}}^{-\left(\text{t}-\text{TD2}/\dot{V}\text{O2}\tau \text{2on}\right)}\right]\times U_{\text{2}}$$

where *U*_1_ = 0, when time (*t*) is less than the time delay (TD) 1 from the onset of exercise; *U*_1_ = 1, for *t* = TD; *U*_2_ = 0, for *t* < TD_2_; and *U*_2_ = 1, for *t* = TD_2_; $\dot{V}{\text{O}}_{\text{2}}$ is the $\dot{V}{\text{O}}_{\text{2}}$ prior to the onset of the rest-to-exercise transition (l), Ampl and Ampl_2_ are the asymptotic amplitudes for the first and second exponential terms (l), respectively; $\dot{V}O_{2}\tau_{\text{on}}$ and $\dot{V}O_{2}\tau_{\text{2on}}$ are the time constants of each exponential (s); and TD and TD_2_ represent the TDs of each equation (s). Since the phase 1–phase 2 transition occurred ≈15–20 s after exercise onset in all participants (visual examination), the initial cardiodynamic component was excluded by deleting the first 20 s of data; the primary component parameters were not distorted by any early cardiodynamic influence (Whipp et al., 2005). A bi-exponential model was used when a significant gain of variance was found for the fit between modeled and measured $\dot{V}{\text{O}}_{\text{2}}$ data in comparison with a mono-exponential model. However, for comparison with the literature (Dupont et al., 2005; Rampinini et al., 2009; Buchheit, 2012b; Buchheit et al., 2012a), data from the first exponential only ($\dot{V}O_{2}\tau_{\text{on}}$ MOD) were retained for analysis.

For the supramaximal exercise, we used a semi-logarithmic model to analyze the $\dot{V}{\text{O}}_{\text{2}}$ on-transient kinetics (Figure 2, mid panel) (Hughson et al., 2000). The application of the semi-logarithmic model to the analysis of $\dot{V}{\text{O}}_{\text{2}}$ data is made possible by the following assumptions: (a) during supramaximal exercise, $\dot{V}{\text{O}}_{\text{2}}$ projects to an unreachable (i.e., above $\dot{V}{\text{O}}_{\text{2}}$max) value; (b) such predicted $\dot{V}{\text{O}}_{\text{2}}$ ($\dot{V}{\text{O}}_{\text{2}}$pred) is proportional to the metabolic power requirement of the task; (c) the rate of the increase in $\dot{V}{\text{O}}_{\text{2}}$ is proportional to the instantaneous difference between $\dot{V}{\text{O}}_{\text{2}}$pred and actual $\dot{V}{\text{O}}_{\text{2}}$ ($\dot{V}O_{2}(t)$), i.e., to the so-called error signal. In order to perform the semi-logarithmic, curve-fitting approach, the natural logarithm of the instantaneous difference (Δ$\dot{V}O_{2}(t)$) between $\dot{V}{\text{O}}_{\text{2}}$pred and the $\dot{V}O_{2}(t)$) values measured at any time was plotted as a function of time. $\dot{V}{\text{O}}_{\text{2}}$pred was extrapolated on the basis of the previously determined individual Δ$\dot{V}{\text{O}}_{\text{2}}$/ΔWR relationship at steady state (pre-HIT warm-up runs, see above). Thereafter, a linear fitting was applied to data points between the TD of the primary component and the TD of the slow component obtained using a bi-exponential function as described above (Equation 2). The time constant of the primary component of $\dot{V}{\text{O}}_{\text{2}}$ ($\dot{V}O_{2}\tau_{\text{on}}$ SEV) was then calculated as the reciprocal of the slope of Δ$\dot{V}O_{2}(t)$) as a function of time. As the time constant calculated with the semilogarithmic model strongly depends on the value of $\dot{V}{\text{O}}_{\text{2}}$pred, we tried to estimate the effect of a potential error in this parameter on the calculated $\dot{V}O_{2}\tau_{\text{on}}$ SEV. In line with Hughson et al. (2000), we allowed a variation of ±5% of $\dot{V}{\text{O}}_{\text{2}}$pred. Therefore, we calculated the slope of the semi-logarithmic relationship when $\dot{V}{\text{O}}_{\text{2}}$pred was 125 and 115% of $\dot{V}{\text{O}}_{\text{2}}$max.

Finally, $\dot{V}{\text{O}}_{\text{2}}$ off-transient kinetics following the supramaximal exercise were modeled for the first 500 s using a bi-exponential function as described above (Equations 1, 2), where $\dot{V}O_{2}\tau_{\text{off}}$ SEV is the time constant of the exponential (s) (Figure 2, lower panel).

### Maximal accumulated o_2_ deficit

To describe the individual $\dot{V}{\text{O}}_{\text{2}}$/speed relationship, the average (visit 3 and 4) $\dot{V}{\text{O}}_{\text{2}}$ measured during last 2 min of the runs at 75%, 85% and 95% of GET was used (Figure 1). All $\dot{V}O_{\text{2p}}$/speed relationships were very large, with *r* = 0.99 ± 0.01. The MAOD was then calculated during the first supramaximal run as the difference between estimated total O_2_ demand (extrapolated from the linear $\dot{V}O_{\text{2p}}$/speed relationship) and measured total O_2_ uptake (Bosquet et al., 2008).

### Near-infrared spectroscopy measurements

The portable NIRS apparatus (Portamon, Artinis, Medical System, Zetten, The Netherlands) used in this study is a two-wavelength continuous wave system, which simultaneously uses the modified Beer–Lambert and spatially resolved spectroscopy methods. The procedure used to collect data was the same as described previously with a similar portable device (Buchheit et al., 2009). Changes in tissue oxyhemoglobin (HbO_2_) and deoxyhemoglobin (HHb) were measured using the differences in absorption characteristics of light at 750 and 850 nm. The difference between HbO_2_ and HHb [Hb_diff_ = (HbO_2_ – HHb)/2] was also calculated (van Beekvelt et al., 2002; Buchheit et al., 2010a). Given the uncertainty of the proton pathlength at rest and during exercise, we used an arbitrary value for the differential pathlength of 3.83 (Delorey et al., 2005). The values for Hb_diff_ are reported as a change from baseline (30 s averaging before each test) in micromolar (μM) units. The use of Hb_diff_ was considered, since it has been shown to be a relevant alternative to HHb when blood flow is not constant; muscle oxygen consumption estimated from Hb_diff_ being more reliable than values estimated from the other NIRS variables (van Beekvelt et al., 2002). We paid great attention to probe replacement. With the portable devices used, firmly attached to the body, there are no moving optical fibers that could cause signal disturbance. NIRS devices were positioned on the vastus lateralis (VL_Hbdiff_) and biceps femoris (BF_Hbdiff_) muscles of the dominant leg used when changing direction, approximately 10 cm from the knee joint and along the vertical axis of the thigh. A surgical marker was used to mark the probe placement for accurate repositioning. The probe and the skin were covered with black tape to prevent contamination from ambient light. Skinfold thickness at the site of application of the NIRS probe was determined before the testing sessions using Harpenden skinfold calipers (British Indicators Ltd, UK). The calculated value of skin and subcutaneous tissue thickness was less than half the distance between the source and the detector. During all tests, the two NIRS systems were connected to a personal computer by Bluetooth for data acquisition (10 Hz), analogue-to-digital conversion, and subsequent analysis.

### Electromyography measurement

EMG data were collected from the dominant leg, using an eight channel Datalog EMG system (Biometrics DataLOG P3X8, Gwent, UK). The contracted muscle belly of the BF and gastrocnemius medial (MG) were identified. Before placing the electrodes, the overlying skin was carefully prepared. The hair was shaved, and the skin was lightly abraded to remove the outer layer of epidermal cells and thoroughly cleansed with alcohol to reduce the skin-electrode interface impedance. Biometrics SX230 active (Ag/AgCl) electrodes separated by 2 cm were carefully taped to the belly of each muscle, parallel to the muscle fibers, using hypoallergenic adhesive tape and cotton wool swabs to minimize sweat induced interference. A passive reference electrode (Biometrics R300) was placed on the pisiform bone of the wrist, with its wiring passed through participants' t-shirt to allow free running movements and to restrict its impedance. The EMG device was secured and fixed to a waist belt. To prevent movement artefact, wires between the electrodes and the device were secured to the skin with adhesive tape and leads braided to minimize electromagnetically induced interference. Signals were sampled at 1000 Hz, amplified (1000x), band-pass filtered (20–450 Hz), and stored for offline analysis on a 512-Mb MMC flashcard (Biometrics DataLOG P3X8; Gwent, UK). Data were imported from the Biometrics unit in 1-ms increments into Spike 2 version 5 (Cambridge Electronics Design, Cambridge, UK) and saved for offline analysis. The data were smoothed using route mean squared (RMS) analysis, which was calculated for a 50-ms window.

EMG data (μv) were calculated for each step (active contraction). Since a minimum of three strides of EMG data per subject provide information as reliable as that obtained from twelve strides during gait trials (Arsenault et al., 1986), we analyzed five strides from the middle of each run, with similar peak amplitudes (Albertus-Kajee et al., 2011). Onset and offset of muscle activity were determined as a deviation greater than two standard deviations (SD) from the mean of three 50-ms windows of inactivity (Allison et al., 1993). The fastest 22-m sprint performed before HIT was analyzed by isolating five peak amplitude contractions from the middle of the sprint recording. The resultant mean amplitudes were averaged and used for the normalization, i.e., the EMG data during HIT were thereby expressed as a percentage of the EMG measured during the fastest 22-m sprint (Albertus-Kajee et al., 2011).

### Blood lactate measurement

Three minutes after the end of all tests and immediately after each set during HIT, a fingertip blood sample (5 μL) was collected and blood [La] concentration was determined with a Lactate Pro analyzer (Arkray Inc, Kyoto, Japan). The analyzer was calibrated with supplied standards prior to each test. The suitability and reproducibility of this analyzer has been previously established throughout the physiological range of 1.0–18.0 mmol.L^−1^ (Pyne et al., 2000).

### Statistical analyses

Data are presented as means and SD. Data in the text and figures are presented as means with 90% confidence limits (CL) and confidence intervals (CI), respectively. All data were first log-transformed to reduce bias arising from non-uniformity error. While this was not initially designed, four players could not complete more than two sets of HIT (see “Results”). Therefore, in addition to the correlation analyses based on the whole group data, additional analyses were carried out on these two subgroups, constituted *a posteriori*: players who did not manage to run more than two sets (*n* = 4, <3 sets group), and players who completed at least five sets (*n* = 7, ≥5 sets group). Data were then analyzed for practical significance using magnitude-based inferences (Hopkins, 2007; Hopkins et al., 2009). We used this qualitative approach because traditional statistical approaches often do not indicate the magnitude of an effect, which is typically more relevant to athletic performance than any statistically significant effect. Between-group standardized differences or effect sizes (90% CI) in the selected anthropometric, physiological and performance variables were calculated using pooled SD. Threshold values for Cohen effect size statistics were >0.2 (small), >0.5 (moderate), >0.8 (large), and >1.2 (very large). Probabilities were also calculated to establish whether the true (unknown) differences were lower, similar or higher than the smallest worthwhile difference or change (0.2 multiplied by the between-subject SD, based on Cohen's effect size principle). Since it is not the absolute ASR that is determinant for HIT performance/tolerance, but rather its amplitude in relation to v$\dot{V}{\text{O}}_{\text{2}}$max, between-group comparisons in ASR were further adjusted for difference in v$\dot{V}{\text{O}}_{\text{2}}$max (Hopkins, 2007). For instance, an unfit player with a poor v$\dot{V}{\text{O}}_{\text{2}}$max but a high MSS would display a large ASR, which is unrealistically beneficial for HIT tolerance. Conversely, it is intuitive that for a given v$\dot{V}{\text{O}}_{\text{2}}$max, a greater ASR (as a results of a greater MSS) may be beneficial for HIT tolerance. Additionally, because of the strong influence of adipose tissue thickness (ATT, i.e., fat + skin layer) on the changes in NIRS variables during exercise (van Beekvelt et al., 2001), between-group comparisons in VL_Hbdiff_ and BF_Hbdiff_ were adjusted for difference in ATT [calculated as 1/2 of the skinfold thickness at the site of application of the NIRS (van Beekvelt et al., 2001)] (Hopkins, 2007). Quantitative chances of higher or lower differences were evaluated qualitatively as follows: <1%, almost certainly not; 1–5%, very unlikely; 5–25%, unlikely; 25–75%, possible; 75–95%, likely; 95–99%, very likely; and >99%, almost certain. If the chance of higher or lower differences was >5%, the true difference was assessed as unclear. Otherwise, we interpreted that change as the observed chance. Pearson's correlation coefficients were calculated (SPSS 19, SPSS Inc, Chicago, USA) to establish the respective relationships between physiological responses to HIT and individual characteristics (e.g., v$\dot{V}{\text{O}}_{\text{2}}$max, MSS, time constant of oxygen kinetics). Because v$\dot{V}{\text{O}}_{\text{2}}$max and $\dot{V}{\text{O}}_{\text{2}}$max are important determinants of fatigue during HIT (Rampinini et al., 2008; Dupont et al., 2010a), correlations including $\dot{V}{\text{O}}_{\text{2}}$ kinetics, jumping performance and EMG data were also adjusted for v$\dot{V}{\text{O}}_{\text{2}}$max and $\dot{V}{\text{O}}_{\text{2}}$max using partial correlations. Similarly, correlations including NIRS variables were adjusted for ATT using partial correlations (van Beekvelt et al., 2001). The magnitude of correlation [*r* (90% CL)] between test measures were assessed with the following thresholds: <0.1, trivial; = 0.1–0.3, small; <0.3–0.5, moderate; <0.5–0.7, large; <0.7–0.9, very large; and <0.9–1.0, almost perfect. If the 90% CI overlapped positive and negative values, the magnitude was deemed unclear; otherwise the magnitude was deemed to be the observed magnitude (Hopkins et al., 2009).

## Results

### Completion of the standardized hit protocol

While we expected all players to complete the eight sets of the present HIT protocol, four players stopped exercise during the third set because of exhaustion. Similarly, two players stopped at the end of the fifth set, while the remaining seven other managed to complete the eight sets (Table 1).

**Table 1:** **Anthropometric, physiological and performance characteristics for the team sport players with respect to the number of HIT sets completed before exhaustion**.

	**<3 sets**	**≥5 sets**	**Standardized differences**	**Rating**	**Chances for greater/similar/lower values for the ≥5 sets group compared with the <3 group**
*n*	4	7			
Age (year)	30.0 ± 1.4	30.5 ± 4.4	0.1 (−0.8; 1.1)	Unclear	46/29/25
Height (cm)	177 ± 6	182 ± 7	+0.6 (−0.5; 1.7)	Unclear	76/14/10
Body mass (kg)	84.7 ± 7.0	79.2 ± 6.4	−0.8 (−0.9; 0.3)	Unclear	7/12/82
Training volume (hr.week^−1^)	2.2 ± 0.5	6.0 ± 1.8	+3.8 (2.8; 4.9)	Very large	100/0/0
(game excluded)					
$\dot{V}{\text{O}}_{\text{2}}$max (ml.min^−1^.kg^−1^)	52 ± 5	57 ± 7	+0.8 (−0.3; 1.8)	Large	83/12/5
v$\dot{V}{\text{O}}_{\text{2}}$max (km.h^−1^)	14.0 ± 1.1	15.9 ± 1.1	+1.5 (0.4; 2.7)	Very large	97/2/1
MSS (km.h^−1^)	28.8 ± 0.3	29.7 ± 1.1	+1.0 (0.1; 1.9)	Large	93/5/2
ASR (km.h^−1^)	14.7 ± 1.4	13.7 ± 1.8	−0.6 (−0.6; 0.4)	Unclear	9/15/76
ASR^*^ (km.h^−1^)	13.2 ± 0.3	14.5 ± 1.3	+1.2 (0.1; 2.3)	Very large	93/4/3
Supraximal run (s)	139 ± 21	125 ± 31	−0.5 (−1.5; 0.5)	Unclear	11/18/71
MAOD (mlO_2_.min^−1^.kg^−1^)	50 ± 15	52 ± 9	+0.1 (−1.1; 1.3)	Unclear	45/25/30
DJ (cm)	19 ± 8	26 ± 6	+0.9 (−0.5; 2.2)	Unclear	84/8/8
DJ (W)	2956 ± 614	3090 ± 542	+0.2 (−0.9; 1.4)	Unclear	52/23/25
CMJ (cm)	36 ± 1	38 ± 6	+0.5 (−0.4; 1.4)	Unclear	71/19/10
CMJ (W)	3945 ± 263	3822 ± 505	−0.3 (−1.3; 0.7)	Unclear	19/24/57
$\dot{V}O_{2}\tau_{\text{on}}$ MOD (s)	29 ± 14	27 ± 6	−0.2 (−1.5; 1.1)	Unclear	26/24/50
$\dot{V}O_{2}\tau_{\text{on}}$ SEV (s)	31 ± 14	30 ± 6	−0.1 (−1.4; 1.2)	Unclear	33/26/41
$\dot{V}O_{2}\tau_{\text{off}}$ SEV (s)	51 ± 2	48 ± 9	−0.4 (−1.3; 0.5)	Unclear	13/22/65

Mean (SD) values for age, height, body mass, training volume, maximal oxygen uptake ($\dot{V}{\text{O}}_{\text{2}}$max), the speed associated with $\dot{V}{\text{O}}_{\text{2}}$max (v$\dot{V}{\text{O}}_{\text{2}}$max), maximal sprinting speed (MSS), anaerobic speed reserve (ASR), time to exhaustion during the first supramaximal run, maximal accumulated oxygen deficit (MAOD), drop jump performance (DJ), counter movement jump performance (CMJ), $\dot{V}{\text{O}}_{\text{2}}$ kinetics at exercise onset in the moderate-intensity domain ($\dot{V}O_{2}\tau_{\text{on}}$MOD), $\dot{V}{\text{O}}_{\text{2}}$ kinetics at exercise onset in the severe intensity domain ($\dot{V}O_{2}\tau_{\text{on}}$SEV), and $\dot{V}{\text{O}}_{\text{2}}$ kinetics at exercise cessation in the severe intensity domain ($\dot{V}O_{2}\tau_{\text{off}}$SEV).

* Adjusted for v$\dot{V}{\text{O}}_{\text{2}}$max.

### Pulmonary oxygen kinetics

$\dot{V}{\text{O}}_{\text{2}}$ kinetics data are presented in Table 1. Irrespective of the exercise, the coefficients of determination (0.95–0.99) obtained between actual $\dot{V}{\text{O}}_{\text{2}}$ and modeled responses were significant (*P* < 0.001) for all models used to characterize the $\dot{V}{\text{O}}_{\text{2}}$ kinetics. The standard error was 3.8 ± 1.6% and 4.2 ± 1.1% for$\dot{V}O_{2}\tau_{\text{on}}$ MOD and $\dot{V}O_{2}\tau_{\text{off}}$ SEV, respectively. Time to exhaustion during the first and second supramaximal run were 132 ± 23 and 107 ± 12 s, respectively [with a standardized difference of −3.4 (90%CL: −4.1; −2.7)]. Values for $\dot{V}O_{2}\tau_{\text{off}}$ SEV were 31 ± 8, 28 ± 7 and 35 ± 8 s for 120 %v$\dot{V}{\text{O}}_{\text{2}}$max, 115 %v$\dot{V}{\text{O}}_{\text{2}}$max and 125 %v$\dot{V}{\text{O}}_{\text{2}}$max, respectively.

### Correlations

**Figure 3:**
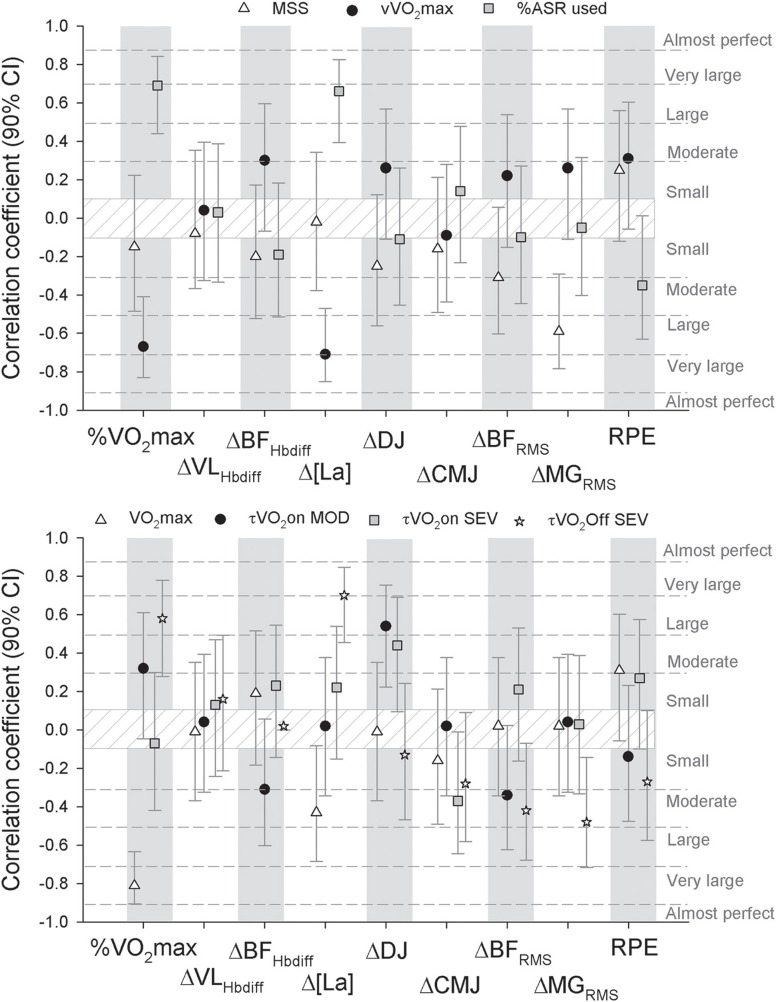
**Correlation coefficients (with confidence intervals) for the relationships between physiological, perceptual and neuromuscular responses to the two first sets of HIT [oxygen uptake expressed as a percentage of maximal oxygen uptake (%$\dot{V}{\text{O}}_{\text{2}}$max), changes in vastus lateralis (ΔVL_Hbdiff_) and biceps femoris (ΔBF_Hbdiff_) oxygenation, changes in blood lactate (Δ[La]), changes in drop jump and counter movement jump height (ΔDJ and ΔCMJ), changes in muscle activity of the biceps femoris (ΔBF_RMS_), medial gastrocnemius (ΔMG_RMS_), and rate of perceived exertion (RPE)] and either locomotor profile [upper panel, maximal spring speed (MSS), the speed associated with maximal oxygen uptake (v$\dot{V}{\text{O}}_{\text{2}}$max), and the percentage of anaerobic reserve used (%ASR)] or measures of aerobic fitness and metabolic control [lower panel, maximal oxygen uptake ($\dot{V}{\text{O}}_{\text{2}}$max), time constants of the primary component of the pulmonary $\dot{V}{\text{O}}_{\text{2}}$ kinetics at exercise onset in the moderate and severe exercise intensity domain, $\dot{V}O_{2}\tau_{\text{on}}$MOD and $\dot{V}O_{2}\tau_{\text{on}}$SEV, respectively and the time constants of the primary component of the pulmonary $\dot{V}{\text{O}}_{\text{2}}$ kinetics after exercise performed in the severe intensity domain, $\dot{V}O_{2}\tau_{\text{off}}$SEV]**.

Figure 3 illustrates the correlation coefficients, in all players pooled together, for the relationships between physiological and neuromuscular responses to the two first sets of HIT and locomotor profile (upper panel) and measures of aerobic fitness and metabolic control (lower panel). Some of the metabolic responses to HIT (i.e., average $\dot{V}{\text{O}}_{\text{2}}$ during HIT and post HIT [La] accumulation) were moderately to very-largely and negatively correlated with v$\dot{V}{\text{O}}_{\text{2}}$max, the % of ASR used, $\dot{V}{\text{O}}_{\text{2}}$max and $\dot{V}O_{2}\tau_{\text{off}}$ SEV, while there was no clear relationship with MSS, MAOD, $\dot{V}O_{2}\tau_{\text{on}}$ MOD or $\dot{V}O_{2}\tau_{\text{on}}$ SEV. Jumping performance responses were unlikely related to players' profile (unclear relationships), except for the moderate-to-large correlations found between changes in DJ and $\dot{V}O_{2}\tau_{\text{on}}$ MOD and $\dot{V}O_{2}\tau_{\text{on}}$ SEV. Changes in neuromuscular activation during HIT were negatively correlated with players' MSS (large correlation for ΔMG_RMS_) and $\dot{V}O_{2}\tau_{\text{off}}$ SEV (moderate correlations for both ΔBF_RMS_ and ΔMG_RMS_). However, when adjusted for either $\dot{V}{\text{O}}_{\text{2}}$max or v$\dot{V}{\text{O}}_{\text{2}}$max, the latter correlations were all rated as unclear. Finally, perceptual responses to HIT were unrelated to any of the selected variables.

### Between-group differences in response to hit

$\dot{V}{\text{O}}_{\text{2}}$ (moderate difference), VL_Hbdiff_ (very large difference), and [La] (large difference) responses to the first two sets of HIT were the only variables that differed between the players which could finish at least five sets or only two (Table 2). When comparing the two groups, there was no clear difference in changes in jumping performance, neuromuscular activation and perceptual responses to the two first sets of HIT (Table 2).

**Table 2:** **Physiological responses to the first two sets of HIT for the team sport players with respect to the number of HIT sets completed before exhaustion**.

	**<3 sets**	≥**5 sets**	**Standardized differences**	**Rating**	**Chances for greater/similar/lower values for the ≥5 group compared with the <3 group**
$\dot{V}{\text{O}}_{\text{2}}$ (%$\dot{V}{\text{O}}_{\text{2}}$max)	87 ± 6	82 ± 10	−0.6 (−1.3; 0.1)	Moderate	3/13/85
ΔVL_Hbdiff_ (μM)	−10 ± 5	−20 ± 7	−1.4 (−3.1; 0.4)	Very large	5/6/89
ΔBF_Hbdiff_ (μM)	−12 ± 2	−16 ± 4	−0.90 (−2.5; 0.6)	Unclear	10/11/79
Δ[La] (mmol.l^−1^)	7.7 ± 2.3	4.8 ± 3.5	−1.1 (−1.7; −0.4)	Large	0/2/98
ΔDJ (%)	−8.3 ± 11.1	−6.2 ± 8.1	+0.2 (−0.6; 1.0)	Unclear	51/31/18
ΔCMJ (%)	−2.9 ± 6.2	−3.2 ± 7.1	0.0 (−0.8; 0.7)	Unclear	29/36/35
ΔBF_RMS_ (%)	−6.4 ± 7.1	−10.6 ± 8.8	−0.5 (−1.5; 0.6)	Unclear	13/19/68
ΔMG_RMS_ (%)	−8.7 ± 3.1	−9.0 ± 13.6	0.0 (−0.9; 1.0)	Unclear	38/30/32
RPE (6–20)	14.6 ± 2.8	14.7 ± 2.1	0.1 (−0.7; 0.9)	Unclear	39/34/27

Mean (SD) values for oxygen uptake ($\dot{V}{\text{O}}_{\text{2}}$max), changes blood lactate accumulation (Δ[La]), changes in vastus lateralis (ΔVL_Hbdiff_) and biceps femoris (ΔBF_Hbdiff_) oxygenation, changes in drop (ΔDJ) and countermovement (ΔCMJ) jump height, and changes in muscle activity of the biceps femoris [ΔBF_RMS_] and medial gastrocnemius [ΔMG_RMS_] and rate of perceived exertion (RPE).

### Between-group differences in players' locomotor and fitness profile

Finally, players that managed to run at least five sets of HIT (i.e., ≥5 sets group), were, in comparison to those who could not complete more than two (i.e., <3 sets group), fitter (largely to very-largely greater $\dot{V}{\text{O}}_{\text{2}}$max and v $\dot{V}{\text{O}}_{\text{2}}$max), faster (largely greater MSS) and have a very largely greater training load at the time of the study (Table 1). There was, however, no clear difference between the two groups with respect to neuromuscular performance, or $\dot{V}{\text{O}}_{\text{2}}$ kinetics. When ASR and $\dot{V}{\text{O}}_{\text{2}}$ kinetics were adjusted for differences in v$\dot{V}{\text{O}}_{\text{2}}$max, players in the ≥ 5 sets group displayed a very largely greater ASR and a largely slower $\dot{V}O_{2}\tau_{\text{off}}$ SEV (Table 1).

## Discussion

In the present study, we compared for the first time the respective impact of selected measures of aerobic fitness ($\dot{V}{\text{O}}_{\text{2}}$max), metabolic control ($\dot{V}{\text{O}}_{\text{2}}$ kinetics at exercise onset and cessation in the moderate and severe intensity domains) and locomotor function (i.e., MSS and v$\dot{V}{\text{O}}_{\text{2}}$max) on players' tolerance to a standardized HIT running in team sport players (i.e., as inferred from the acute metabolic, neuromuscular and perceptual responses to HIT). The main findings of the present study were as follows: (1) metabolic responses to the first two sets of HIT were, in all participants, negatively correlated with (in order of importance) $\dot{V}{\text{O}}_{\text{2}}$max, v$\dot{V}{\text{O}}_{\text{2}}$max, % of ASR used, and $\dot{V}O_{2}\tau_{\text{off}}$ SEV, while there was no clear relationship with MSS, $\dot{V}O_{2}\tau_{\text{on}}$ MOD or $\dot{V}O_{2}\tau_{\text{on}}$ SE, (2) the impairment in neuromuscular activation during HIT was negatively correlated with players' MSS and $\dot{V}O_{2}\tau_{\text{off}}$ SEV, (3) pulmonary $\dot{V}{\text{O}}_{\text{2}}$, muscle oxygenation and [La] responses to the first two sets of HIT were the only variables that differed between the players who could finish at least five sets and those which could not complete more than two sets, and (4) players that managed to run at least five sets of HIT, were, in comparison to those which could not complete more than two sets, fitter, faster and have a very largely greater training load at the time of the study; there was, however, no clear difference between the two groups with respect to baseline jumping power or $\dot{V}{\text{O}}_{\text{2}}$ kinetics.

### Physiological, perceptual, and neuromuscular performance responses to high-intensity intermittent running in team sport players

While the present analyses (correlations analysis and between-group comparisons), given the limited sample size, must be considered with care, they may offer researchers a starting point toward understanding how cardiorespiratory fitness, metabolic control (i.e., $\dot{V}{\text{O}}_{\text{2}}$ kinetics) and locomotor function may/may not influence tolerance to HIT in an homogenous groups of team sport players. It is, however, worth mentioning that the present group sample size (*n* = 11) were similar to those in Dupont's studies [i.e., 11 (Dupont et al., 2005) and 12 (Dupont et al., 2010b), respectively]. More importantly, we used Hopkins' scale to interpret both the between-group differences and the magnitude of the correlations, which is well suited for the analysis of data obtained with low sample sizes (Hopkins et al., 2009).

The standardized HIT protocol designed for the present study (22-m runs to be performed in 4 s) elicited high physiological and perceptual responses (as inferred from $\dot{V}{\text{O}}_{\text{2}}$, Hb_diff_, [La], RPE responses during the two first sets), which were similar to those previously reported for almost similar HIT protocols, e.g., $\dot{V}{\text{O}}_{\text{2}}$ values >80% $\dot{V}{\text{O}}_{\text{2}}$max, ΔHb_diff_ > 10 μ M, Δ[La] > 7 mmol.L^−1^, and RPE >7 (CR10 Borg's scale) during repeated 4-s sprint sequences (Buchheit, 2010). The acute ~3 (CMJ) to ~7 (DJ) % decrease in jumping performance observed in the present study following HIT is also consistent with the substantial decrements in CMJ height reported after repeated-sprint sequences in team sport players [−8% after 6 25-m sprints, (Buchheit, 2010)] and track sessions in 400-m runners [5–10% for 60- to 100-m sprints (Gorostiaga et al., 2010)]. This likely impaired neuromuscular performance during/following HIT was concomitant to the progressively decreased RMS during the two first sets (ΔBF_RMS_ and ΔGM_RMS_), which is consistent with a progressive impairment in muscle activation during such repeated high-speed efforts (Mendez-Villanueva et al., 2008). In overall, this important level of physiological and neuromuscular strain was likely too high for four players (36%), who could not complete the eight sets of the present HIT protocol (Tables 1, 2). While this was not initially designed since all players were expected to complete the eight sets, this allowed the constitution *a posteriori* of two subgroups for further analysis (see “Methods”). The following paragraphs will highlight the physiological and locomotor factors that were the most likely to affect HIT tolerance, both for all players pooled together (correlation analyses) and between each group (between-group comparisons).

### Metabolic and locomotor determinants of hit tolerance

In the present study, metabolic responses to HIT were very-largely (and negatively) correlated with $\dot{V}{\text{O}}_{\text{2}}$max, v$\dot{V}{\text{O}}_{\text{2}}$max, and % of ASR used (Figure 3). Additionally, in comparison to the players who could not complete more than two sets, the players who managed to run at least five sets presented lower $\dot{V}{\text{O}}_{\text{2}}$ and [La] responses to the first two sets, and have a very likely greater muscle deoxygenation (Table 2). This greater muscle deoxygenation responses in the ≥5 sets group (Table 2) is consistent with the greater muscle O_2_ extraction capacity observed in fit/trained individuals (Bailey et al., 2009), and might have assisted these latter players to rely less on anaerobic metabolisms during HIT. This may have, in turn, improved their HIT tolerance (this hypothesis is partly confirmed by the lower blood [La] accumulation in this group, Table 2). Taken together, present results suggest therefore that the relative metabolic load reached after the first two sets was likely the main factor determining HIT tolerance in this population. This was also confirmed by the fact that players in the ≥5 sets group presented, in comparison with the <3 group, very largely greater v$\dot{V}{\text{O}}_{\text{2}}$max, ASR and training volume, and largely $\dot{V}{\text{O}}_{\text{2}}$max and MSS (Table 1); there was, however, no clear difference in MAOD. The more “successful” players also tended to be lighter and skinnier (although we did not measure whole % body fat) and to jump higher (despite no difference when consider jumping power). While it could appear paradoxical that MAOD did not discriminate successful and less successful players with respect to HIT tolerance (despite the supramaximal nature of the HIT protocol), the relative importance of this latter measure compared with $\dot{V}{\text{O}}_{\text{2}}$max, v$\dot{V}{\text{O}}_{\text{2}}$max, and % of ASR used might be too low. For instance, the impact of MAOD on HIT tolerance might only become significant when considering players with similar $\dot{V}{\text{O}}_{\text{2}}$max and v$\dot{V}{\text{O}}_{\text{2}}$max (Midgley et al., 2007a).

Taken together, these results confirm the importance of cardiorespirtaory fitness for improved HIT tolerance/performance (Rampinini et al., 2008; Dupont et al., 2010a), but also highlight the importance of a player's locomotor profile (i.e., v$\dot{V}{\text{O}}_{\text{2}}$max and MSS). In the present study, blood [La] accumulation response to HIT (Δ[La]) shared more variance with v$\dot{V}{\text{O}}_{\text{2}}$max (*r* = −0.71 (−0.85; −0.47); *r*^2^ = 50%) than with $\dot{V}{\text{O}}_{\text{2}}$max (*r* = −0.43 (−0.68; −0.08); *r*^2^ = 18%) (Figure 3). This is likely related to the fact that v$\dot{V}{\text{O}}_{\text{2}}$max integrates, in addition to cardiorespiratory fitness, players' running economy (Di Prampero et al., 1986). For the aforementioned reason, v$\dot{V}{\text{O}}_{\text{2}}$max generally shows greater association with distance running (Paavolainen et al., 1999) and repeated-sprint performance (Buchheit, 2012b) than $\dot{V}{\text{O}}_{\text{2}}$max. Also, in addition to v$\dot{V}{\text{O}}_{\text{2}}$max, the value of MSS has also to be considered with respect to HIT tolerance. While MSS is unlikely to directly impact on the metabolic responses to HIT (there was no clear correlation, Figure 3), it also determines the proportion of the ASR used during HIT. A lower use of the ASR likely prevents excessive local peripheral physiological disturbance, thereby sparing anaerobic capacity and neuromuscular function, in turn allowing for the maintenance of high-intensity running capacity (Bundle et al., 2003). In the present study, %ASR used was largely to very-largely negatively correlated with both $\dot{V}{\text{O}}_{\text{2}}$ and [La] response to HIT (Figure 3). In practical terms, training priorities should therefore be determined with respect to these two locomotor entities for improved HIT tolerance. For example, a player with an already high MSS but moderate v$\dot{V}{\text{O}}_{\text{2}}$max would be advised to improve v$\dot{V}{\text{O}}_{\text{2}}$max. Conversely, emphasis on MSS development could be advised in players displaying an already high v$\dot{V}{\text{O}}_{\text{2}}$max and/or in population likely more responsive to this type of work (i.e., young players).

Finally, it is also worth noting that the impairment in neuromuscular activation (ΔMG_RMS_) during HIT was negatively correlated with players' MSS (Figure 3). This is consistent with previous findings on neuromuscular activity during repeated-sprint sequences, where faster/more powerful players generally experience greater neuromuscular adjustments (Girard et al., 2011). In the present study, however, changes in muscle EMG were not related to HIT tolerance (i.e., there was no between-group difference in either ΔBF_RMS_ or ΔMG_RMS_, Table 2). The fact that changes in muscle activity had no functional consequences may be related to both the nature of the task [in contrast to cycling (Mendez-Villanueva et al., 2008), performance during sprint running can be maintained via adjustments in stride parameters and intra-muscle coordination] and the fact that the importance of metabolic factors likely overpowered that of neuromuscular adjustments in the present population and exercise protocol.

### Pulmonary $\dot{V}{\text{O}}_{\text{2}}$max kinetics and hit tolerance

In the present study, all $\dot{V}{\text{O}}_{\text{2}}$ transitions could be correctly modeled by either mono-, bi-exponential or semi-logarithmic functions (95% CI ranged from 2 to 5%), which confirms the accuracy of the present measures. When appropriate, the use of a bi-exponential model ($\dot{V}O_{2}\tau_{\text{on}}$ MOD and $\dot{V}O_{2}\tau_{\text{off}}$ SEV) was designed to avoid any distortion of the primary component parameter by a possible $\dot{V}{\text{O}}_{\text{2}}$ slow component (Whipp et al., 2005). However, for comparison with the literature [i.e., (Dupont et al., 2005; Rampinini et al., 2009; Buchheit, 2012b; Buchheit et al., 2012a) for $\dot{V}O_{2}\tau_{\text{on}}$ MOD and for (Dupont et al., 2010b) $\dot{V}O_{2}\tau_{\text{off}}$ SEV], only data from the first exponential were retained for analysis. Values for $\dot{V}O_{2}\tau_{\text{on}}$ MOD (Table 1) were very similar to those of previously described for adult team sport players (Dupont et al., 2005; Rampinini et al., 2008, 2009; Buchheit, 2012b). In contrast, present $\dot{V}O_{2}\tau_{\text{off}}$ SEV values tended to be greater than those reported by Dupont et al. (2010b). Differences in the studied populations (age, sport, and training background), as well as $\dot{V}{\text{O}}_{\text{2}}$ kinetics modeling technique may however account for these differences. This is to our knowledge, however, the first time that the kinetics of $\dot{V}{\text{O}}_{\text{2}}$, at exercise onset, as measured in the severe intensity domain, were put in relation to HIT running performance. This is an important point since in previous studies, only transitions from rest to moderate exercise intensities were used to derive the on-$\dot{V}{\text{O}}_{\text{2}}$ kinetics (Dupont et al., 2005; Rampinini et al., 2009; Buchheit, 2012b); inferences to metabolic control during HIT exercise were therefore limited. Present $\dot{V}O_{2}\tau_{\text{on}}$ SEV values (i.e., 50 s) were within the range of these previously reported at a similar exercise intensity in sedentary [40 s (Hughson et al., 2000) and 60 s (Adami et al., 2011)] and recreationally active [72 s (Carter et al., 2006)] males. We acknowledge, however, that the use of a supramaximal exercise to derive $\dot{V}O_{2}\tau_{\text{on}}$ SEV have limitations, since it does not allow assessing a possible slow component in the $\dot{V}{\text{O}}_{\text{2}}$ response, which is actually likely to be observed during HIT running exercise. Future studies should therefore be conducted to assess the possible relationship between the $\dot{V}{\text{O}}_{\text{2}}$ slow component responsiveness during heavy intensity (but not supramaximal) exercise and the acute metabolic, neuromuscular and perceptual responses to HIT.

In the present population, we found no correlation between any of the on-$\dot{V}{\text{O}}_{\text{2}}$ kinetics measures and physiological or perceptual response to HIT. Additionally, we found no difference in on-$\dot{V}{\text{O}}_{\text{2}}$ kinetics (either as measured in the moderate or the severe intensity domain) between the players that managed to run at least five sets and those which could not complete more than two. From a physiological view point, the lack of association between response to HIT and $\dot{V}O_{2}\tau_{\text{on}}$ could be related to the fact that the ability to repeat high-intensity efforts might depend more on immediate between-sprints recovery mechanisms [i.e., PCr resynthesize and ion transport (Girard et al., 2011)] than on a possibly reduced O_2_ deficit at exercise onset (Jones and Burnley, 2009). Present results confirm that the importance of on-$\dot{V}{\text{O}}_{\text{2}}$ kinetics for HIT tolerance might be overestimated (Buchheit, 2012a,b). Finally, it is also worth mentioning that, and especially in the case of a simple investigation on the relationship between two variables (Dupont et al., 2005, 2010b), researchers are generally more prone to publish positive results only (i.e., publication bias phenomenon). In these lines, whether “team sport players should train to improve $\dot{V}{\text{O}}_{\text{2}}$ kinetics *per se*” (Rampinini et al., 2009) still remains to be examined with longitudinal interventions. Additionally, the training method that might have the greatest potential to accelerate $\dot{V}{\text{O}}_{\text{2}}$ kinetics has still to be defined (Berger and Jones, 2007; Bailey et al., 2009; McKay et al., 2009).

In the present study, however, despite no between-group differences in off-$\dot{V}{\text{O}}_{\text{2}}$ kinetics (as measured in the severe intensity domain, Table 1), we found large correlations between $\dot{V}O_{2}\tau_{\text{off}}$ SEV and $\dot{V}{\text{O}}_{\text{2}}$ and [La] response to HIT, and the decrease in neuromuscular activation (ΔMG_RMS_) following HIT. The fact that the players with slower off-$\dot{V}{\text{O}}_{\text{2}}$ kinetics displayed the highest $\dot{V}{\text{O}}_{\text{2}}$ responses is not surprising and consistent with previous findings in cyclists during repeated sprints (Buchheit et al., 2012a) (i.e., the slower the kinetics, the greater the time spent at high $\dot{V}{\text{O}}_{\text{2}}$ levels). The large correlation between $\dot{V}O_{2}\tau_{\text{off}}$ SEV and both [La] response and changes in neuromuscular activation is consistent with the belief that a faster post-efforts replenishment of muscle O_2_ may accelerate PCr resynthesize (Borsheim and Bahr, 2003), lowering anaerobic glycolytic system contribution (as evidenced by the large correlation with Δ[La], Figure 3) and in turn, improving successive high-intensity exercise tolerance/performance (Girard et al., 2011). Present results are therefore partly in agreement with the positive association reported by Dupont et al. (2010b) between $\dot{V}O_{2}\tau_{\text{off}}$ SEV and repeated-sprint ability (the faster the $\dot{V}O_{2}\tau_{\text{off}}$ recovery, the lower the speed decrement index).

The lack of a between-group difference in $\dot{V}O_{2}\tau_{\text{off}}$ SEV (Table 1), despite the significant correlation between $\dot{V}O_{2}\tau_{\text{off}}$ SEV and some responses to HIT, may be due the fact that differences in $\dot{V}{\text{O}}_{\text{2}}$max and v$\dot{V}{\text{O}}_{\text{2}}$max were likely more determinant than off-$\dot{V}{\text{O}}_{\text{2}}$ kinetics for HIT tolerance. In support to this hypothesis, once adjusted for $\dot{V}{\text{O}}_{\text{2}}$max or v$\dot{V}{\text{O}}_{\text{2}}$max, the latter correlations between $\dot{V}O_{2}\tau_{\text{off}}$ SEV and [La] or MG_RMS_ responses were not clear anymore. Within the context of the present study, impairments in muscle activation (e.g., ΔMG_RMS_) might not be the primary cause of exercise tolerance (Girard et al., 2011). Additionally, off-$\dot{V}{\text{O}}_{\text{2}}$ kinetics might not accurately reflect muscle metabolisms (Krustrup et al., 2009), especially during successive sprints (Buchheit et al., 2012a). Whilst muscle energy turnover consistently declines rapidly after exercise in the muscle, successive sprints might progressively increase systemic O_2_ utilization (e.g., ventilation and cardiac work, thermoregulation, and gluconeogenesis), thereby further dissociating local from systemic measurements. In support of this idea, Krustrup et al. (2009) did not find a relationship between pulmonary and muscle $\dot{V}{\text{O}}_{\text{2}}$ recovery kinetics following either low- or high-intensity exercise. Taken together, present results and data from the literature suggest that off-$\dot{V}{\text{O}}_{\text{2}}$ kinetics, despite their moderate association with changes in muscle activation during HIT running exercise (Figure 3), may not be the primary variables determining acute metabolic, neuromuscular and perceptual responses to HIT, and, in turn, tolerance to HIT.

In conclusion, present results confirm the importance of aerobic fitness ($\dot{V}{\text{O}}_{\text{2}}$max), and locomotor function (i.e., MSS and v$\dot{V}{\text{O}}_{\text{2}}$max) for improved HIT tolerance in team sport players. In contrast, with respect to the acute physiological and perceptual responses to HIT observed in the present population, the importance of metabolic control ($\dot{V}{\text{O}}_{\text{2}}$ kinetics, irrespective of the exercise intensity domains considered in the present study) appears limited. Future studies should however consider the possible impact of the $\dot{V}{\text{O}}_{\text{2}}$ slow component responsiveness during heavy intensity (but not supramaximal) exercise on HIT tolerance. Present results also suggest that from a practical point of view, training strategies targeting the development of locomotor function (i.e., high-intensity training and/or neuromuscular-oriented work) should be implemented in priority to improve HIT tolerance.

### Conflict of interest statement

The authors declare that the research was conducted in the absence of any commercial or financial relationships that could be construed as a potential conflict of interest.
